# PIGMENTED BASAL CELL CARCINOMA SUCCESSFULLY TREATED WITH 5% IMIQUIMOD CREAM

**DOI:** 10.4103/0019-5154.43204

**Published:** 2008

**Authors:** Vandana Mehta, C Balachandran

**Affiliations:** *From the Department of Skin and STD, Kasturba Medical College, Manipal*

**Keywords:** *Basal cell carcinoma*, *pigmented*, *treatment*, *Imiquimod*

## Abstract

Basal cell carcinoma (BCC) is the most common malignant skin tumor, amongst which the nodular, nodulo ulcerative and superficial types comprise nearly 80% of all BCCs. Topical Imiquimod, an immune response modifier has been found to be effective in superficial and nodular types of BCC with histological clearance rates of up to 100%. We report our experience of treatment a large pigmented BCC on the face with topical Imiquimod 5% cream.

## Introduction

Basal cell carcinoma (BCC) is the most common malignant skin tumor. Though surgical excision, curettage with or without electrocautery, cryotherapy and intralesional interferons are all available as potential treatments for BCC, they are painful and require a skilled medical practitioner. Topical imiquimod, an immune response modifier has been found to be effective in superficial and nodular types of BCC with histological clearance rates of up to 100%. We report our experience of treatment a large pigmented BCC on the face with topical Imiquimod 5% cream.

## Case History

**Fig. 1:**
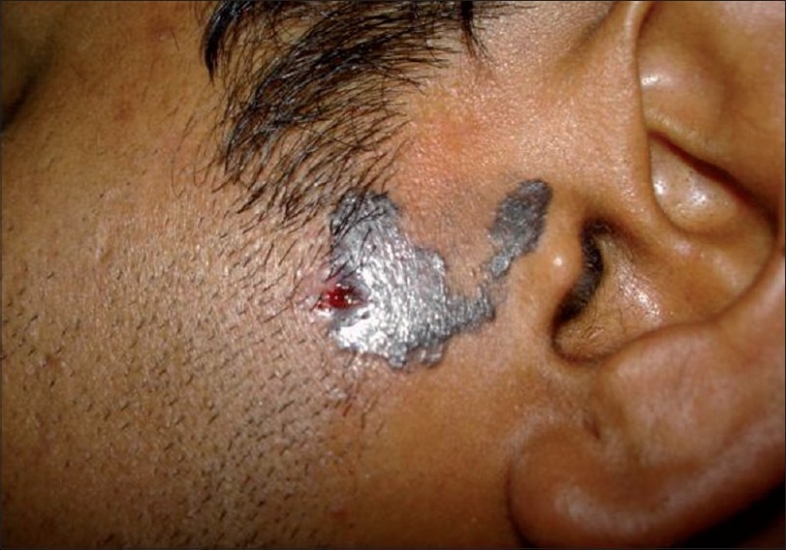
Hyperpigmented plaque with raised borders

**Fig. 2:**
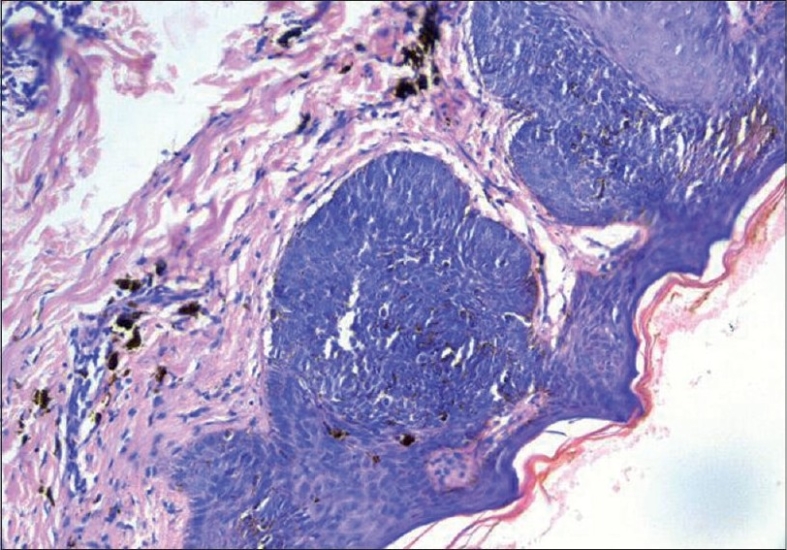
Photomicrograph showing nests of basaloid cells in continuity with the epidermis 20

**Fig. 3:**
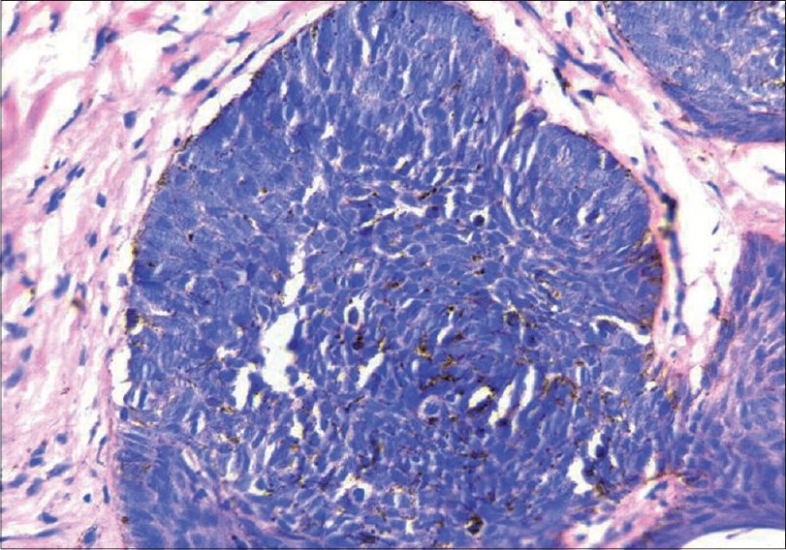
Photomicrograph showing close up of basaloid cells with peripheral palisading 40×

**Fig. 4:**
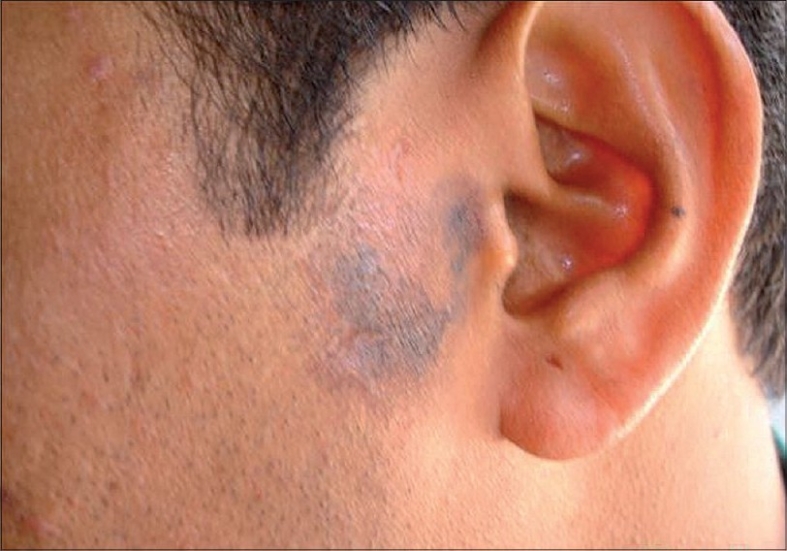
Post-treatment photograph showing resolution of pigmentation from the periphery

A 37-year-old male presented with an asymptomatic, hyperpigmented plaque on his left cheek of 8 years duration. The lesion was small at onset and had gradually increased to its present state. There was no history of exposure to radiation other than routine sun exposure. There were no systemic symptoms. Cutaneous examination revealed a solitary well-defined hyperpigmented plaque measuring 3 × 4 cm with raised pigmented margins near the left ear (Fig. 1). There was no ulceration or regional lymphadenopathy. Systemic examination was normal. A clinical diagnosis of pigmented basal cell carcinoma was made. Biopsy from the edge of the plaque showed multiple nests of basaloid cells with peripheral palisading in the papillary dermis in continuity with the overlying epithelium (Figs. 2 and 3). Our patient was started on Imiquimod 5% cream to be applied three times a week on alternate days. After four weeks of application, he developed mild erythema with burning sensation over the lesion, which subsided in due course. At12 weeks there was near complete resolution of the pigmentation from the periphery with only some central activity (Fig 4). The treatment was further continued for four weeks after which there was complete clearance. A repeat biopsy done at this stage showed no residual tumor on multiple sections.

## Discussion

Imiquimod, a heterocyclic imidazoquinoline amide, is an immune response modifier that has both antitumor and antiviral activity. It was first approved by the US Food and Drug Administration in 1997 for the treatment of external genital and perianal warts, and in 2004 for the treatment of actinic keratoses and superficial basal cell carcinoma.^1^ Besides these there are also reports of its use in non genital warts, molluscum contagiosum, Bowen's disease, squamous cell carcinoma, extra mammary Paget's disease, keratoacanthoma, Mycosis fungoides, morphea, prevention of keloids after surgery, infantile hemangiomas, porokeratosis of Mibelli, cutaneous leishmaniasis, actinic cheilitis, recurrent genital herpes and eccrine poroma.^2^

Imiquimod as a monotherapy has shown cure rates ranging from 70% to 100% with twice daily, once daily and thrice weekly regimens for both superficial and nodular BCC.^3^ Clinical and histological cure rate of 100% was seen with twice daily application of Imiquimod for a period of six weeks.^4^

The precise mechanism of action of Imiquimod in BCC is unknown. It has been postulated that ultraviolet radiation induces mutations in the tumor suppressor genes and alters the immunosurveillance, so that tumor cells escape from apoptosis and cytotoxic T cells. The Th2 cytokines that down regulate tumor surveillance are raised in BCC. Imiquimod is a Toll like receptor (TLR) 7 agonist that acts on the immune system by stimulating the monocytes, macrophages and dendritic cells to produce interferon alpha, Th1 cytokines (IL-1, IL-6, IL-10, IL-12), tumor necrosis factor a and G-CSF, thereby counteracting Th2 cytokines and promoting tumor surveillance.^5^ It also enhances the activity of natural killer cells and epidermal Langerhan's cells.

Although imiquimod is considered safe, both systemic and local side-effects have been reported.^6^ Most of the local side-effects have been application site reactions such as burning, itching, pain, erythema, erosions and hypopigmentation which may be seen with any dosing regimen, but typically increase in frequency and severity as the frequency of application increases. Systemic side-effects are usually mild and include headache, fever, malaise, myalgia, arthralgia, nausea and diarrhea. Our patient continued treatment for 16 weeks and experienced only minimal application site reactions which subsided is due course.

For patients who have failed to respond to other therapeutic modalities or who have had recurrence following standard therapy or who are medically unfit for surgery, Imiquimod may be beneficial.
